# Integrating endogeneity in survey sampling using instrumental-variable calibration estimator

**DOI:** 10.1016/j.heliyon.2024.e33969

**Published:** 2024-07-04

**Authors:** Muhammad Nadeem Intizar, Muhammad Ahmed Shehzad, Haris Khurram, Soofia Iftikhar, Aamna Khan, Abdul Rauf Kashif

**Affiliations:** a Department of Statistics, Bahauddin Zakariya University, Multan, Pakistan; b Department of Mathematics and Computer Science, Faculty of Science and Technology, Prince of Songkla University, Pattani Campus, Pattani, Thailand; c Department of Sciences and Humanities, National University of Computer and Emerging Sciences, Chiniot-Faisalabad Campus, Chiniot, Pakistan; d Department of Statistics, Shaheed Benazir Bhutto Women University Peshawar, Pakistan; e Institute of Southern Punjab Multan, Pakistan

**Keywords:** Endogeneity, Instrumental variables, Auxiliary variables, Calibration estimator, Survey sampling

## Abstract

The endogeneity problem arises when the auxiliary variables correlate to the error terms. In such cases, appropriate instrumental variables ensure efficient estimation. Calibration has recognized itself as an important methodological tool at a large scale to estimate the population total in survey sampling. Which does not offer efficient estimation in the presence of endogeneity. When endogeneity is present in the auxiliary variables, the calibration using endogenous auxiliary variables may produce biasedness and increase variance due to inappropriate model assumptions. In this article, we propose instrumental-variable calibrated estimators by using the classical instrumental-variables approach for the case of exact identification that are more efficient than conventional calibration estimators when some auxiliary variables are endogenous. The necessary properties of the proposed estimators are presented. Our study is backed by both the simulation study and a real data example to check the performance of the proposed estimators.

## 1 Introduction

Estimation of population total or means has significance while considering the survey data. Various researchers have proposed different estimators to estimate population total and mean under different sampling designs and by considering different problems in survey data. Liu and Arslan [1] proposed the estimators for population mean using auxiliary proportions. Ahmad et al. [2] suggested the generalized estimators for population mean. Wang et al. [3] Derived estimators for population mean by simple and double sampling in situations of extreme values. The calibration technique was derived by Deville and Särndal [4] to obtain an estimator of the population total using some sample weights called calibrated weights. These weights are obtained by minimizing the distance to the Horvitz-Thompson weights $\left(d_{k}=\frac{1}{\pi_{k}}\right)$ with the condition on the calibration equations to be satisfied. The resulting weights will be a function of the auxiliary variables.

Suppose we wish to estimate the total of the variable of interest Y in a finite population $U=\left\{Y_{1}\ldots ,{\;Y}_{k},\ldots ,{\;Y}_{N}\right\}$. A probability sample s is selected from the population with sampling designp(s), and $y_{k}$ is the value of *k*-th unit of the study variable Y for all k∈s (complete response) with a known inclusion probability $\pi_{k}>0$ for each element *k*, and the corresponding sampling design weight ${\;d}_{k}$. A vector of *p* auxiliary variables $X_{k}^{T}=\left(x_{k1},\ldots ,x_{kj},\ldots ,x_{kp}\right)$ is the transposed vector whose elements are the values of the auxiliary variables for the kth unit associated with $y_{k}$. We observe ($y_{k}$, $X_{k})$ for the elements k∈s. The population total of X is $t_{x}=\sum_{U}X_{k}$ is known and Horvitz-Thompson estimators is ${\hat{t}}_{x\pi }=\sum_{s}d_{k}X_{k}\text{.}$ Deville and Särndal [4] suggested the calibration estimator defined in equation (1.1) as

(1.1) $${\hat{t}}_{yw}=\sum_{s}w_{k}y_{k}$$

where $w_{k}$ weights selected to satisfy

$$\sum_{s}w_{k}X_{k}=\sum_{U}X_{k}$$

To minimize the distance between the design weights ${\;w}_{k}$ and initial weights ${\;d}_{k}$, any distance function $G_{k}$ suggested by Deville and Särndal [4] can be minimized under some basic conditions with constraints given in eq. (1.2). Thus, calibration weights are linear functions of design weights and available auxiliary information. If $\lambda =\left\{\lambda_{1},\ldots ,\lambda_{j},\ldots ,\lambda_{p}\right\}$ Langrange multipliers vector. Then the Lanragian equation can be written as equation (1.2).

(1.2) $$\sum_{s}d_{k}G\left(w_{k},d_{k}\right)-\lambda \left(\sum_{s}w_{k}X_{k}-\sum_{U}X_{k}\right)=0$$

So $\varphi_{s}\left(\lambda \right)=\left(t_{x}-{\hat{t}}_{x\pi }\right)$ and λ can be found by the method of Newton's optimization discussed in equation (1.3) as:

(1.3) $$\lambda_{v+1}=\lambda_{v}+{\left\{\varphi_{s}\left(\lambda_{v}\right)\right\}}^{-1}\left\{t_{x}-{\hat{t}}_{x\pi }-\varphi_{s}\left(\lambda_{v}\right)\right\}$$

so the value of λ is

$$\lambda ={\left(\sum_{s}d_{k}q_{k}X_{k}{\overset{´}{X}}_{k}\right)}^{-1}\left(t_{x}-{\hat{t}}_{x\pi }\right)$$

Hence we get the calibrated weights in equation (1.4) as:

(1.4) $$w_{k}=d_{k}F\left(q_{k}x_{k}^{T}\lambda \right)=d_{k}F\left(u\right)$$

where $u=q_{k}{\overset{´}{x}}_{k}\lambda$.

The proposed calibrated weights gave the different results for different distance functions. Deville and Särndal [4] suggested different distance functions. The chi square distance function gave the class of calibrated weights such as

(1.5) $$w_{k}=d_{k}\left(1+q_{k}x_{k}^{T}\lambda \right)$$

where $q_{k}$ in equation (1.5) is the parameter that can be chosen to for improved calibrated weights and relative efficiency. Estevao and Särndal [5] used arbitrary positive value of $q_{k}$ to improve the calibrated estimator. Which is the same as the generalized regression estimator (GREG) proposed by Cassel et al. [6] and the obtained estimator can be deduced as a model-based and design-based estimator Cardot et al., 2017. [7].

(1.6) $${\hat{t}}_{GREG}=\sum_{s}w_{k}y_{k}={\hat{t}}_{x\pi }+{\hat{b}}_{s}\left(t_{x}-{\hat{t}}_{x\pi }\right)$$

where ${\hat{b}}_{s}={\left(\sum_{s}d_{k}q_{k}x_{k}^{T}x_{k}\right)}^{-1}\sum_{s}d_{k}q_{k}x_{k}^{T}y_{k}$ in equation (1.6). However, this minimum distance technique in calibration offers almost identical estimators for different distance functions. For studying the properties of calibrated estimators, Estevao and Särndal [5] suggested calibration estimators under two-phase sampling. Shehzad [8] and Goga and Shehzad [9] produced the penalized calibrated estimators. Shehzad et al. [10] and Brirah et al. [11] proposed modified calibration methods for estimating the population total. Alam and Hanif [12] proposed cosmetic calibration estimators. Kott [13], Kott [14], Särndal [15], and Kim (2010) also used the calibration technique for different conditions to derive the calibrated estimators. Park and Kim [16] proposed model-based instrumental-variable calibrated estimators to minimize the anticipated variance in calibration estimator also used under two-phase sampling. Endogeneity is a classical problem which arises due to the correlation between the independent variables and error terms. Wooldridge [17] suggested to use an instrumental variable $Z_{k}$. Which are highly correlated with each endogenous component of X but independent of e to deal the problem of endogeneity. In survey data, the problem of endogeneity also arises when we model the data to estimate the population total. When endogeneity is present in the auxiliary variables, the calibration using endogenous auxiliary variables may produce biasedness and increase variance due to inappropriate model assumptions. This estimation problem has not been addressed in calibration estimation.

In this paper, we proposed the instrumental-variable calibration estimator using model-assisted and model-based approaches when some auxiliary variables are endogenous. The mathematical properties of the proposed estimator were verified, and the performance of the proposed estimator was evaluated using a simulation study and real data. In sections 3, 4, properties of proposed estimators are presented. In section 5, the performance of the estimators has been evaluated by a simulation study and a real data example.

## 2 Instrumental variables (IV) regression

One of the most important assumptions of the Classical Linear Regression Model (CLRM) is that the regressors are exogenous. The violation of this assumption $Cov\left(X_{i},e_{i}\right)\neq 0\text{,}$ that is, the regressors are correlated with the error term, is called Endogeneity. The solution to this violation is the method of Instrumental-variables (IV). An estimator for which the endogenous and instrumental variables are the same is referred to as just or exact identified. An estimator for which the instrumental variables are more than the endogenous variables is called the over-identified estimator [18]. Wright [19] first introduced instrumental variables and used them to estimate supply and demand elasticity for butter and flaxseed. Reiersøl [20] applied the same method in the context of errors-in-variables models in his dissertation. Let $X=\left(X_{1},{\;X}_{2},\ldots ,{\;X}_{p}\right)$ be a nn×p matrix of known regressors and suppose the following super population regression model.

(2.1) Y=Xβ+e

Y is a (n×1) vector of the dependent variable, and X is (n×p) non-random matrix of independent variables. Also $\left(X^{T}X\right)$ is a full-rank matrix and e is a (n×1) vector of residuals also assumed that the expected value of e is zero and $e_{p}$ are uncorrelated. The variance of e is constant (homoscedastic), i.e. $,var\left(e\right)=\sigma^{2}I$, also assumed that X and e are independent, i.e. cov(X,e)=0. It means that the explanatory variables are exogenous and β is (n×1) vector of unknown parameters. Then the ordinary least square (OLS) estimator is

$${\hat{\beta }}_{OLS}={\left(X^{T}X\right)}^{-1}X^{T}y$$

The ordinary least square estimator ${\hat{\beta }}_{OLS}$ is unbiased and has minimum variance such as

$$var.cov\left({\hat{\beta }}_{OLS}\right)=E\left[\left(y-X{\hat{\beta }}_{OLS}\right){\left(y-X{\hat{\beta }}_{OLS}\right)}^{T}\right]={\hat{\sigma }}^{2}{\left(X^{T}X\right)}^{-1}$$

Hence ${\hat{\beta }}_{OLS}$ is an unbiased and consistent estimator of β. On the other hand, when X and e are correlated, that is cov(X,e)≠0, it means that the explanatory variable X is endogenous then the OLS estimator is biased and inconsistent. In this situation, it is good to use the estimates to predict the value of the dependent variable given the value of X. However, the estimate does not recover the causal effect of X on y. So, to estimate the parameter β consider a set of variables Z (instrumental variables) which are highly correlated with each endogenous component of X but independent of e[17]. If the relationship between each endogenous component of $x_{i}$ and the instrument is defined in equation (2.2) and given as:

(2.2) $${\;x}_{i}=Z_{i}\delta +u_{i}$$

Then the instrumental variable (IV) estimator is

(2.3) $${\hat{\beta }}_{IV}={\left(Z^{T}X\right)}^{-1}Z^{T}y$$

Instrumental–variable estimator ${\hat{\beta }}_{IV}$ in equation (2.3) is unbiased and consistent under certain regularity conditions.

## 3 Instrumental-variable calibration approach

The calibration approach is usually used without assuming the super population model [4]. The calibration technique consists of estimating the population total $\left(\;t_{y}=\sum_{i}^{N}Y_{i}\right)$ such as

$${\hat{t}}_{yw}=\sum_{s}W_{k}y_{k}$$

with constraint in equation (1.2) i.e.

$${\;W}_{s}^{T}X_{s}=1_{u}X$$

The distance function (chi-square distance) is

$${\;\left(W_{s}-d_{s}\right)}^{T}{П}_{s}\left(W_{s}-d_{s}\right)$$

where ${П}_{s}=diag\left(q_{k}^{-1}d_{k}^{-1}\right)$. Then Lagrange multiplier is

(3.1) $$L={\left(W_{s}-d_{s}\right)}^{T}{П}_{s}\left(W_{s}-d_{s}\right)-\lambda \left(W_{s}^{T}X_{s}-1_{u}X\right)=0$$

So taking derivatives of L co ncerningw in equation (3.1) we obtained the value of λ. By putting the value of λ we finally get the weights as:

$$w_{s,c}=d_{s}+{П}_{s}^{-1}X_{s}{\left(X_{s}^{T}{{П}_{s}^{-1}X}_{s}\right)}^{-1}{\left({1^{T}}_{u}X-d_{s}^{T}X_{s}\right)}^{T}$$

(3.2) $$w_{s,c}=d_{s}+\left(1_{u}^{T}X-d_{s}^{T}X_{s}\right){\left(X_{s}^{T}{{П}_{s}^{-1}X}_{s}\right)}^{-1}{X_{s}}^{T}{П}_{s}^{-1}$$

hence the calibration estimator of $t_{y}$ using equation (3.2) becomes

$${\hat{t}}_{y}={w_{s,c}}^{T}y_{s}=d_{s}^{T}y_{s}+\left(1_{u}^{T}X-d_{s}^{T}X_{s}\right){\left(X_{s}^{T}{{П}_{s}^{-1}X}_{s}\right)}^{-1}{X_{s}}^{T}{П}_{s}^{-1}y_{s}$$

We propose the instrumental-variable calibration estimator by the instrumental-variable calibration approach proposed by Ref. [5] without using the distance minimum function approach such as

$${\hat{t}}_{yw}=W_{k}y_{k}$$

where $W_{k}$ is the calibrated weight obtained by the instrumental-variable approach subject to

$${\;W}_{s}^{T}X_{s}=1_{u}X$$

The weight with unknown λ is

(3.3) $${\;W}_{s}=d_{s}+\lambda {П}_{s}^{-1}Z_{s}$$

where ${\;П}_{s}=diag\left(q_{k}^{-1}d_{k}^{-1}\right)$, $q_{k}$ is a positive integer in the present study, we take ${\;q}_{k}=1$, and $Z_{s}$ is the sample restriction of Z, the classical instrumental variable used instead of the endogenous auxiliary variable. By plugging in the weights in the calibration constraint we find the value of λ as

$$\lambda ={\left({Z_{s}}^{T}{П}_{s}^{-1}X\right)}^{-1}\left(1_{u}X-d_{s}X_{s}\right)$$

Put the value of λ in equation (3.3) weights equation and finally, we get the required weights as

(3.4) $$W_{IVC}=d_{s}+{П}_{s}^{-1}Z_{s}{\left({Z_{s}}^{T}{П}_{s}^{-1}X\right)}^{-1}\left(1_{u}X-d_{s}X_{s}\right)$$

so, the instrumental-variable calibration estimator for the total $t_{y}$ by using equation (3.4) is as:

$${\hat{t}}_{IVC}=W_{s,IVC}y_{s}=\left(d_{s}+\left(1_{u}^{T}X-d_{s}^{T}X_{s}\right){\left(Z_{s}^{T}{{П}_{s}^{-1}X}_{s}\right)}^{-1}{Z_{s}}^{T}{П}_{s}^{-1}\right)y_{s}$$

$$=d_{s}^{T}y_{s}+\left(1_{u}^{T}X-d_{s}^{T}X_{s}\right){\left(Z_{s}^{T}{{П}_{s}^{-1}X}_{s}\right)}^{-1}{Z_{s}}^{T}{П}_{s}^{-1}y_{s}$$

(3.5) $${\hat{t}}_{IVC}=d_{s}^{T}y_{s}+\left(1_{u}^{T}X-d_{s}^{T}X_{s}\right){\hat{\beta }}_{IVC}$$

where ${{\hat{\beta }}_{IVC}=\left(Z_{s}^{T}{{П}_{s}^{-1}X}_{s}\right)}^{-1}{Z_{s}}^{T}{П}_{s}^{-1}y_{s}$. The estimator ${\hat{t}}_{IVC}$ defined in equation (3.5) is a model-assisted (designed-based) instrumental-variable estimator.

### 3.1 Properties of model-assisted instrumental-variable calibration estimator

Some properties of the model-assisted Instrumental-variable calibration estimator $({\hat{t}}_{IVC}$) are presented and their proof are available in appendix.

Theorem 1 *The model-assisted Instrumental-variable calibration estimator* (${\hat{t}}_{IVC})$ *is biased*, *and its biases are given by*

$$\text{Bias}\;\left({\hat{t}}_{IVC}\right)=-E\left(\sum_{s}\frac{y_{k}}{\pi_{k}}-\sum_{U}X_{k}^{T}\right){\hat{\beta }}_{IVC}$$

*where*

$${\hat{\beta }}_{IVC}={\left(Z_{k}^{T}{П}_{s}^{-1}X_{s}\right)}^{-1}Z_{s}^{T}{{П}_{s}^{-1}y}_{s}$$

Theorem 2 *The asymptotic variance of the instrumental-variable model-assisted calibration estimator*$({\hat{t}}_{IVC}$ ) *is given by*

$$MSE\left({\hat{t}}_{IVC}-t_{y}\right)={E\left({\hat{t}}_{IVC}-t_{y}\right)}^{2}=Var\left({\hat{t}}_{IVC}\right)+{\left(Bias\left({\hat{t}}_{IVC}\right)\right)}^{2}$$

*if* ${\hat{\beta }}_{IVC}-\beta_{OLS}=o_{p}\left(1\right)$ *then the asymptotic variance of* ${\hat{t}}_{IVC}$ *is*

$$Var\left({\hat{t}}_{IVC}\right)=\sum_{U}\sum_{U}\Delta_{kl}\left(\frac{y_{k}-X_{k}{\hat{\beta }}_{IV}}{\pi_{k}}\right)\left(\frac{y_{l}-X_{l}{\hat{\beta }}_{IV}}{\pi_{l}}\right)$$

## 4 Model-based instrumental-variable calibration approach

Usually, without the auxiliary information, ${\hat{t}}_{y}$ is determined by the Horvitz-Thompson [21] estimator, defined as

(4.1) $${\hat{t}}_{y}=d_{k}^{T}y_{k}$$

The estimator in equation (4.1) may be improved by using the auxiliary variables in the form of model-based estimation. A model identified the set of conditions that describe a class of distribution of $Y=\left\{y_{1},y_{2},\ldots ,y_{N}\right\}$ [22]. Kumar et al. [23] proposed the model-based calibration estimator when the study and auxiliary variables are inversely related. We propose a model-based instrumental-variable calibration estimator of Y by the Instrumental-variable calibration approach proposed by Ref. [5] under the model given in equation (2.1) as:

Y=Xβ+e

which does not satisfy the assumption of exogeneity, that is $E\left(x_{i},e_{i}\right)\neq 0$. We propose a model-based instrumental-variable calibration estimator of Y as:

(4.2) $${\hat{t}}_{IVMBC}=W_{s}y_{S}$$

where $W_{s}$ calibrated weights which are obtained by the instrumental-variable calibration technique. Subject to the constraint

$$W_{s}^{T}X_{s}=1_{u}X$$

Since $X_{s}$ is endogenous, we use instrumental-variable $Z_{s}$ instead of endogenous auxiliary variables. By using the Instrumental-variable calibration approach proposed by Ref. [5], the weights in equation (4.2) become

(4.3) $${\;W}_{s}=1_{s}+\lambda Z_{s}$$

Plug in the value of weight in equation (3.1) we get $\left(1_{s}+\lambda Z_{s}\right)X_{s}=1_{u}X$. By solving it we find the value of λ as $\lambda =1_{u-s}X{\left(Z_{s}^{T}X_{s}\right)}^{-1}$. Plug in the value of λ in equation (4.3) final weights are as:

(4.4) $$W_{s}=1_{s}+{1_{u-s}X\left(Z_{s}^{T}X_{s}\right)}^{-1}Z_{s}^{T}$$

thus, the proposed Instrumental-variable model-based calibration estimator of $t_{y}$ using equation (4.4) becomes

$${\hat{t}}_{MBIVC}=W_{s}y_{S}=\left(1_{s}+{1_{u-s}X\left(Z_{s}^{T}X_{s}\right)}^{-1}Z_{s}^{T}\right)y_{S}$$

$${\hat{t}}_{MBIVC}{=1}_{s}y_{S}+{1_{u-s}X\left(Z_{s}^{T}X_{s}\right)}^{-1}Z_{s}^{T}y_{S}$$

which is equivalent to the

$${\hat{t}}_{MBIVC}{=1}_{s}y_{s}+1_{u-s}X{\hat{\beta }}_{MBIVC}$$

where ${\hat{\beta }}_{MBIVC}={\left(Z_{s}^{T}X_{s}\right)}^{-1}Z_{s}^{T}y_{S}$. So, the model-based instrumental-variable calibration weights ($W_{s}$) perform a similar character to the calibrated weights under certain conditions.

### 4.1 Properties of model-based instrumental-variable calibration estimators

Some Properties of the model-based Instrumental-variables calibration estimator ${\hat{t}}_{IVMBC}$ are presented as theorems.

Theorem 3 *The model-based Instrumental-variable calibration estimator*${\hat{t}}_{IVMBC}$*is biased*, *and its bias is given as*

$$Bias\left({\hat{t}}_{MBIVC}\right)=E\left({\hat{t}}_{MBIVC}-t_{y}\right)=\sum_{U-s}\left(X_{s}^{T}\right)E\left({\hat{\beta }}_{MBIVC}-\beta_{OLS}\right)$$

*where*${\hat{\beta }}_{MBIVC}={\left(Z_{s}^{T}X_{s}\right)}^{-1}Z_{s}^{T}y_{s}$.

Theorem 4 *The Mean Square Error of the model-based Instrumental-variable calibration estimator*${\hat{t}}_{IVMBC}$*is given as*

$$MSE\left({\hat{t}}_{MBIVC}\right)=E{\left({\hat{t}}_{MBIVC}-t_{y}\right)}^{2}=E\left[(\sum_{U-s}X_{k}^{T}{\left(\sum_{s}Z_{k}^{T}X_{k}\right)}^{-1}\left(\sum_{s}(z_{k}\left(y_{k}-X_{k}\beta_{OLS}\right){\left(y_{k}-{\;X}_{k}\beta_{OLS}\;\right)}^{T}z_{k}^{T}\right){\left(\sum_{s}Z_{k}^{T}X_{k}\right)}^{-1}\left(\sum_{s}X_{k}\right)\right]$$

## 5 Simulation scheme

In this section, we draw the empirical results to check the efficiency of the estimators by the Monte Carlo simulation. The present simulation study generates a finite population of size N=1000. For this population, 20 variables $X=\left(X_{1},{\;X}_{2},\ldots ,{\;X}_{20}\right)$ of size 1000 (X matrix is of dimension 1000×20) were generated using normal distribution, in which some are adjusted to have correlation with error terms using a linear function. In this way, they are endogenous. The finite population is based on the pair ($y_{k},x_{k}$) such that $x_{k}$ and $y_{k}$ are linearly related, and the relation obtains the variable of interest Y as defined in equation (2.1). The value of β is taken as 1. The total value of Y which is $t_{y}$ assumed to be the true population total. Instrumental auxiliary variables were also generated using normal distribution but with the assumption that they are correlated with auxiliary variable are unrelated to error terms. A sample of size n=25,50,75,100,150,200,250,300,and350 were taken using Simple Random Sampling without Replacement (SRSWOR) for each draw. Different number of endogenous variables (E), E=1,2,and3, were considered by using a linear model so the error terms relate to corresponding auxiliary variables. Then each endogenous variables replaced by the Instrumental-variable Z, generated to be independent of the error term and correlated with its endogenous auxiliary variable. The number of simulations was R=1000 and generated data were kept fixed in each simulation. All the computational work was done in R language.

### 5.1 Performance evaluation

The performance evaluation of the proposed estimators with conventional estimator is presented using following measures.

Bias: which is calculated for estimated total (${\hat{t}}_{y}$) such as:

$$Bias=E\left({\hat{t}}_{y}\right)-t_{y}$$

Mean Square Error (MSE): which is calculated for estimated total (${\hat{t}}_{y}$) such a

$$MSE=\frac{1}{R}\sum_{i=1}^{R}{\left({\hat{t}}_{iy}-t_{y}\right)}^{2}$$

### 5.2 Simulation result

The results are presented in Table 1, Table 2, Table 3, Table 4, Table 5. These results show the behaviour of all the considered estimators: the HT estimator, GREG or conventional calibration estimator, and Instrumental-Variable Calibration (IVC) estimator for different endogenous auxiliary variables for 20 total auxiliary variables for different sample sizes by (SRSWOR). For every table, the performance of each estimator is examined with two properties Bias and Mean Square Error (MSE).

**Table 1:** Monte Carlo Bias and Mean Square Error (MSE) with one endogenous variable (E=1) i.e. $X_{1}$ is endogenous.

Sample Size	Estimator	Bias	MSE
	HT	−16.361	873393.000
25	GREG	−778.672	621246.000
	IVC	−778.658	621221.000
	HT	−1.190	425585.000
50	GREG	−382.337	156225.000
	IVC	−382.333	156224.000
	HT	−4.971	257230
75	GREG	−245.274	65207.400
	IVC	−245.27	65204.100
	HT	−2.504	190901.000
100	GREG	−183.93	36814.400
	IVC	−183.93	36813.300
	HT	−13.283	125723.000
150	GREG	−114.260	14322.300
	IVC	−114.260	14322.200
	HT	−4.841	93744.400
200	GREG	−80.432	7159.710
	IVC	−80.434	7159.450
	HT	−6.553	71821.200
250	GREG	−60.615	4079.350
	IVC	−60.618	4079.140
	HT	−1.483	53143.100
300	GREG	−47.477	2508.530
	IVC	−47.479	2508.430
	HT	−0.832	42642.500
350	GREG	−37.613	1582.320
	IVC	−37.614	1582.230

**Table 2:** Monte Carlo Biases and Mean Square Error (MSE) with two endogenous variables (E=2) i.e. $X_{1}$ and $X_{2}$ are endogenous.

Sample Size	Estimator	Bias	MSE
	HT	−11.045	1042904.000
25	GREG	−778.750	621374.000
	IVC	−778.730	621333.000
	HT	−1.195	532269.000
50	GREG	−382.420	156285.000
	IVC	−382.420	156285.000
	HT	−4.619	237769.000
100	GREG	−183.960	36833.000
	IVC	−183.960	36832.800
	HT	−15.471	151008.000
150	GREG	−114.260	14326.400
	IVC	−114.260	14326.100
	HT	−8.883	111323.000
200	GREG	−80.434	7160.840
	IVC	−80.437	7160.200
	HT	−6.965	82023.700
250	GREG	−60.598	4078.120
	IVC	−60.599	4078.000
	HT	−2.243	60966.400
300	GREG	−47.473	2509.380
	IVC	−47.474	2509.330
	HT	−0.406	50913.400
350	GREG	−37.601	1581.900
	IVC	−37.601	1581.870

**Table 3:** Monte Carlo Bias and Mean Square Error (MSE) with two endogenous variables (E=2) i.e. $X_{9}$ and $X_{10}$ are endogenous.

Sample Size	Estimator	Bias	MSE
	HT	−11.489	615217.000
50	GREG	−383.490	156733.000
	IVC	−383.350	156646.000
	HT	−2.277	358471.000
80	GREG	−231.530	58098.100
	IVC	−231.390	58049.500
	HT	−0.530	284893.000
100	GREG	−181.740	36030.700
	IVC	−181.640	36008.700
	HT	−4.183	175080.000
150	GREG	−114.070	14434.700
	IVC	−114.010	14429.400
	HT	−0.990	129327.000
200	GREG	−79.734	7123.410
	IVC	−79.701	7121.280
	HT	−1.660	98590.300
250	GREG	−59.882	4082.220
	IVC	−59.875	4084.980
	HT	−0.985	75622.400
300	GREG	−46.741	2513.170
	IVC	−46.723	2513.600
	HT	−2.691	59559.900
350	GREG	−37.209	1610.760
	IVC	−37.199	1612.290

**Table 4:** Monte Carlo Bias and Mean Square Error (MSE) with three endogenous variables (E=3) i.e. $X_{1}$, $X_{2}\;and\;X_{3}$ are endogenous.

Sample Size	Estimator	Bias	MSE
	HT	−7.794	1145201.000
25	GREG	−778.819	621504.800
	IVC	−778.757	621387.700
	HT	−10.167	588039.300
50	GREG	−382.498	156348.000
	IVC	−382.494	156340.400
	HT	−4.504	262701.600
100	GREG	−183.999	36853.180
	IVC	−183.995	36850.250
	HT	−10.167	588039.300
150	GREG	−382.498	156348.000
	IVC	−382.494	156340.400
	HT	10.501	124380.400
200	GREG	−80.436	7162.820
	IVC	−80.438	7161.104
	HT	−6.819	91988.110
250	GREG	−60.582	4077.529
	IVC	−60.580	4077.112
	HT	2.155	67202.490
300	GREG	−47.470	2510.695
	IVC	−47.469	2510.488
	HT	−1.049	56529.500
350	GREG	−37.589	1581.869
	IVC	−37.589	1581.771

**Table 5:** Monte Carlo Biases and Mean Square Error (MSE) with three endogenous variables (E=3) i.e. $X_{13}$, $X_{14}\;and\;X_{15}$ are endogenous.

Sample Size	Estimator	Bias	MSE
	HT	−13.856	562018.300
50	GREG	−286.849	91032.490
	IVC	−286.932	91175.830
	HT	−6.788	335671.800
80	GREG	−173.478	33916.380
	IVC	−173.190	33840.500
	HT	−9.501	252110.500
100	GREG	−133.820	20549.370
	IVC	−133.715	20528.650
	HT	−9.495	166003.000
150	GREG	−85.768	8706.890
	IVC	−85.575	8680.110
	HT	−14.421	125141.100
200	GREG	−61.105	4579.970
	IVC	−60.982	4579.366
	HT	−12.313	86838.950
250	GREG	−45.391	2600.937
	IVC	−45.396	2602.538
	HT	−6.048	71820.870
300	GREG	−35.298	1611.601
	IVC	−35.347	1610.860
	HT	−3.512	55557.950
350	GREG	−27.849	1018.425
	IVC	−27.880	1020.369

Table .1 shows the results of HT, GREG, and IVC in the form of Bias and MSE for n=25,50,75,100,150,200,250,300and350. For all the sample sizes and E=1, the Mean Square Error (MSE) of the proposed Instrumental-Variable Calibrated (IVC) estimator is smaller than the HT and GREG estimators. Table 2 shows the results obtained for similar conditions for two endogenous variables, E=2, for different sample sizes. The Mean Square Error (MSE) of HT and GREG is larger than the proposed Instrumental-variable calibrated (IVC) estimator. Table 3 shows the results obtained for similar conditions for E=2. for different sample sizes, the Mean Square Error (MSE) of HT and GREG is larger than the proposed Instrumental-Variable Calibrated (IVC) estimator. Table 4, Table 5 show the results for three endogenous variables, E=3, for different sample sizes in both cases. The Mean Square Error (MSE) of the proposed Instrumental-Variable Calibrated (IVC) estimator is smaller than HT and GREG estimators.

The results show that the proposed Instrumental-Variable Calibrated (IVC) estimator gave the smaller Mean Square Error (MSE) for small and large sample sizes. So Instrumental-Variable Calibrated (IVC) estimator improves the efficiency over conventional Calibration.

### 5.3 Real data example

To compare the proposed estimators with the Horvitz-Thompsons and conventional calibration estimators (GREG estimator). We used a real data example. The data given by Singh et al. [24] is used to evaluate the model performance. The data are freely and publicly accessible for use at: http://www.kiran.nic.in/pdf/Social_Science/elearning/How_to_Test_Endogeneity_or_Exogeneity_using_SAS-1.pdf. Eight variables of size (N = 376) are in the dataset including Min_Tem (Minimum Temperature), Rain (Average Rainfall), Foodgrain_Yield (Yield of food grain), Latitude (Latitude of a particular location), Longitude (Longitude of a particular location), Foodgrain_yld_FD (First difference of Foodgrain_Yield), Min_Tem_FD (First difference of Min_Tem), Rain_FD (First difference of rain), where the Yield of food grain is a dependent variable. The Auxiliary variables are Minimum Temperature and Rain, and the other five variables, Latitude, Longitude, Foodgrain_yld_FD, Min_Tem_FD, and Rain_FD, are selected as instrumental variables. The Auxiliary variable has already endogeneity reported, so we use the instrumental variables instead of the endogenous auxiliary variables to evaluate the model performance. We considered this data as population data and take a sample of size n = 25, 50, 75, 100, 150, 200, and 250 using SRSWOR.

### 5.4 Real data results

Table .6 presents the results of the three estimators and their Bias and Mean Square Error (MSE) for different sample sizes. When the auxiliary variable, Minimum Temperature, is endogenous, the variable Longitude of a particular location is used as an instrumental variable. The results show that the proposed Instrumental-Variable Calibrated (IVC) estimator has a smaller Mean Square Error (MSE) than HT and GREG estimators when there is a problem of Endogeneity present in the dataset in case of exact identification. This shows that the proposed estimator is more efficient than the HT and GREG.

**Table 6:** Real data Average Bias and Average Mean Square Error (MSE) with one endogenous variable.

Sample Size	Estimator	Bias	MSE
	HT	−184.310	1500018504
25	GREG	−2690.505	1437499886
	IVC	−2403.427	1428790986
	HT	−584.414	723613047
50	GREG	−105.868	664012946
	IVC	−33.187	661193157
	HT	−676.414	470786518
75	GREG	−932.468	434993010
	IVC	−915.803	434817082
	HT	−113.956	340054572
100	GREG	−266.031	300480833
	IVC	−240.996	300443340
	HT	−203.112	182342767
150	GREG	−220.419	165836688
	IVC	−181.663	165650642
	HT	−19.471	105476083
200	GREG	−160.463	96909490
	IVC	−149.517	96621549
	HT	−162.380	60052672
250	GREG	−225.584	53665612
	IVC	−220.437	53623346

## 6 Conclusion

In survey sampling, the calibration restrictions are significant. In this paper, the Instrumental-variable calibration technique is used to find the optimum estimators in the presence of the problem of endogeneity. In Monte-Carlo simulation study and real data example, we examined the performance of the proposed estimator for different sample sizes drawn by simple random sampling without replacement from a finite population. The proposed Instrumental-Variable Calibrated (IVC) estimator in terms of Mean Square Error (MSE) is more efficient than HT and GREG estimators under different sample sizes and varying endogenous variables. The proposed estimator is more efficient as sample size increases. The present study is limited to the exact identification means that the number of instrumental variables equals the number of endogenous variables Further investigation of the over-identification problem is the topic of future research.

## CRediT authorship contribution statement

**Muhammad Nadeem Intizar:** Writing – original draft, Validation, Methodology, Investigation, Formal analysis, Data curation. **Muhammad Ahmed Shehzad:** Writing – review & editing, Visualization, Validation, Supervision, Software, Resources, Project administration, Methodology, Investigation, Conceptualization. **Haris Khurram:** Writing – review & editing, Validation, Supervision, Software, Project administration, Methodology, Investigation, Formal analysis. **Soofia Iftikhar:** Visualization, Methodology, Investigation, Formal analysis. **Aamna Khan:** Writing – review & editing, Visualization, Validation, Resources, Investigation, Formal analysis, Data curation. **Abdul Rauf Kashif:** Writing – original draft, Resources, Investigation, Formal analysis.

## Declaration of competing interest

The authors declare that they have no known competing financial interests or personal relationships that could have appeared to influence the work reported in this paper.

## Appendix Proof of Theorems

Proof of Theorem 1 The model-assisted Instrumental-variable calibration estimator (${\hat{t}}_{IVC})$ is given by

$${\hat{t}}_{IVC}=d_{s}y_{s}+\left(1_{u}^{T}X-d_{s}^{T}X_{s}\right){\hat{\beta }}_{IVC}$$

which can be written as

$${\hat{t}}_{IVC}=\sum_{s}\frac{y_{k}}{\pi_{k}}-\left(\sum_{s}\frac{X_{k}^{T}}{\pi_{k}}-\sum_{U}X_{k}^{T}\right){\hat{\beta }}_{IVC}$$

Subtracting both sides by $t_{y}$.

$${\hat{t}}_{IVC}-t_{y}=\sum_{s}\frac{y_{k}}{\pi_{k}}-\left(\sum_{s}\frac{X_{k}^{T}}{\pi_{k}}-\sum_{U}X_{k}^{T}\right){\hat{\beta }}_{IVC}-t_{y}$$

$$=\sum_{s}\frac{y_{k}}{\pi_{k}}-\left(\sum_{s}\frac{X_{k}^{T}}{\pi_{k}}-\sum_{U}X_{k}^{T}\right){\hat{\beta }}_{IVC}-\sum_{U}y_{k}$$

Applying expected value

$$E\left({\hat{t}}_{IVC}-t_{y}\right)=E\left[\sum_{s}\frac{y_{k}}{\pi_{k}}-\left(\sum_{s}\frac{X_{k}^{T}}{\pi_{k}}-\sum_{U}X_{k}^{T}\right){\hat{\beta }}_{IVC}-\sum_{U}y_{k}\right]$$

$$=E\left(\sum_{s}\frac{y_{k}}{\pi_{k}}\right)-E\left(\sum_{s}\frac{X_{k}^{T}}{\pi_{k}}-\sum_{U}X_{k}^{T}\right){\hat{\beta }}_{IVC}-E\left(\sum_{U}y_{k}\right)$$

$$=\sum_{U}y_{k}-E\left(\sum_{s}\frac{X_{k}^{T}}{\pi_{k}}-\sum_{U}X_{k}^{T}\right){\hat{\beta }}_{IVC}-\sum_{U}y_{k}$$

$$Bias\left({\hat{t}}_{IVC}\right)=E\left({\hat{t}}_{\pi ,IVC}-t_{y}\right)=-E\left(\sum_{s}\frac{X_{k}^{T}}{\pi_{k}}-\sum_{U}X_{k}^{T}\right){\hat{\beta }}_{IVC}$$

Proof of Theorem 2 The model-assisted instrumental-variable calibration estimator $({\hat{t}}_{IVC}$) is given by

$${\hat{t}}_{IVC}=\sum_{s}\frac{y_{k}}{\pi_{k}}-\left(\sum_{s}\frac{X_{k}^{T}}{\pi_{k}}-\sum_{U}X_{k}^{T}\right){\hat{\beta }}_{IVC}$$

Subtracting $t_{y}$ on both sides

$${\hat{t}}_{IVC}-t_{y}=\sum_{s}\frac{y_{k}}{\pi_{k}}-\left(\sum_{s}\frac{X_{k}^{T}}{\pi_{k}}-\sum_{U}X_{k}^{T}\right){\hat{\beta }}_{IVC}-t_{y}$$

$$={\hat{t}}_{y\pi }-\left({\hat{t}}_{x\pi }-t_{x}\right){\hat{\beta }}_{IVC}-t_{y}$$

$$={\hat{t}}_{y\pi }-{{\hat{t}}_{x\pi }\beta_{IVC}+t_{x}\hat{\beta }}_{IVC}-t_{y}-t_{x}{\hat{\beta }}_{IV}+t_{x}{\hat{\beta }}_{IV}-{\hat{t}}_{x\pi }{\hat{\beta }}_{IV}+{\hat{t}}_{x\pi }{\hat{\beta }}_{IV}$$

$$=\left({\hat{t}}_{y\pi }-t_{y}\right)-\left({\hat{t}}_{x\pi }-t_{x}\right){\hat{\beta }}_{IV}+t_{x}\left({\hat{\beta }}_{IVC}-{\hat{\beta }}_{IV}\right)-{\hat{t}}_{x\pi }\left({\hat{\beta }}_{IVC}-{\hat{\beta }}_{IV}\right)$$

$$=\left({\hat{t}}_{y\pi }-t_{y}\right)-\left({\hat{t}}_{x\pi }-t_{x}\right){\hat{\beta }}_{IV}-\left({\hat{t}}_{x\pi }-t_{x}\right)\left({\hat{\beta }}_{IVC}-{\hat{\beta }}_{IV}\right)$$

Now

$$\frac{1}{N}\left({\hat{t}}_{IVC}-t_{y}\right)=\frac{1}{N}\left({\hat{t}}_{y\pi }-t_{y}\right)-\frac{1}{N}\left({\hat{t}}_{x\pi }-t_{x}\right){\hat{\beta }}_{IV}-\frac{1}{N}\left({\hat{t}}_{x\pi }-t_{x}\right)\left({\hat{\beta }}_{IVC}-{\hat{\beta }}_{IV}\right)$$

Since

$$\frac{1}{N}\left({\hat{t}}_{x\pi }-t_{x}\right){\hat{\beta }}_{IV}=o_{p}\left(\frac{1}{\sqrt{n}}\right)$$

$${\hat{\beta }}_{IVC}-{\hat{\beta }}_{IV}=o_{p}\left(1\right)$$

So

$$=\left({\hat{t}}_{y\pi }-t_{y}\right)-\left({\hat{t}}_{x\pi }-t_{x}\right){\hat{\beta }}_{IV}$$

$$={\hat{t}}_{y\pi }-t_{y}-{\hat{t}}_{x\pi }{\hat{\beta }}_{IV}+t_{x}{\hat{\beta }}_{IV}$$

$$=\left({\hat{t}}_{y\pi }-{\hat{t}}_{x\pi }{\hat{\beta }}_{IV}\right)-\left(t_{y}-t_{x}{\hat{\beta }}_{IV}\right)$$

and

$$\frac{1}{N}\left(t_{y}-t_{x}{\hat{\beta }}_{IV}\right)=o_{p}\left(1\right)$$

So

$$\left({\hat{t}}_{IVC}-t_{y}\right)=\left({\hat{t}}_{y\pi }-{\hat{t}}_{x\pi }{\hat{\beta }}_{IV}\right)$$

$$MSE\left({\hat{t}}_{IVC}-t_{y}\right)={E\left({\hat{t}}_{\pi ,IVC}-t_{y}\right)}^{2}=Var\left({\hat{t}}_{IVC}\right)+{\left(Bias\left({\hat{t}}_{IVC}\right)\right)}^{2}$$

if $Bias\left({\hat{t}}_{IVC}\right)=0$.

$${E\left({\hat{t}}_{IVC}-t_{y}\right)}^{2}=Var\left({\hat{t}}_{IVC}-t_{y}\right)$$

$$=Var\left({\hat{t}}_{y\pi }-{\hat{t}}_{x\pi }{\hat{\beta }}_{IV}\right)$$

$$=Var\left(\sum_{s}\frac{y_{k}}{\pi_{k}}-\sum_{s}\frac{X_{k}}{\pi_{k}}{\hat{\beta }}_{IV}\right)$$

$$=Var\left(\sum_{s}\frac{y_{k}-X_{k}{\hat{\beta }}_{IV}}{\pi_{k}}\right)$$

$$=Var\left(\sum_{U}\frac{y_{k}-X_{k}{\hat{\beta }}_{IV}}{\pi_{k}}\right)\left(I_{k}\right)$$

$$=\sum_{U}{\left(\frac{y_{k}-X_{k}{\hat{\beta }}_{IV}}{\pi_{k}}\right)}^{2}Var\left(I_{k}\right)+\sum_{k\in U}\sum_{l\in U,J\neq k}\left(\frac{y_{k}-X_{k}{\hat{\beta }}_{IV}}{\pi_{k}}\right)\left(\frac{y_{l}-X_{l}{\hat{\beta }}_{IV}}{\pi_{l}}\right)Cor\left(I_{k}I_{l}\right)$$

$$Var\left({\hat{t}}_{IVC}\right)=\sum_{U}\sum_{U}\Delta_{kl}\left(\frac{y_{k}-X_{k}{\hat{\beta }}_{IV}}{\pi_{k}}\right)\left(\frac{y_{l}-X_{l}{\hat{\beta }}_{IV}}{\pi_{l}}\right)$$

Proof of Theorem 3 The model-based instrumental-variable calibration estimator ${\hat{t}}_{MBIVC}$ is given by

$${\hat{t}}_{MBIVC}=W_{MBIVC}^{T}y_{s}=\sum_{s}y_{k}+\left(\sum_{u-s}X_{k}^{T}\right){\hat{\beta }}_{MBIVC}$$

Subtracting $t_{y}$ on both sides

$${\hat{t}}_{IVC}-t_{y}=\sum_{s}y_{k}+\left(\sum_{u-s}X_{k}^{T}\right){\hat{\beta }}_{MBIVC}-t_{y}$$

now plug the value of $y_{k}$ and ${\;t}_{y}$.

$$=\sum_{s}{(X}_{s}^{T}\beta_{OLS}+e_{k})+\left(\sum_{u-s}X_{k}^{T}\right){\hat{\beta }}_{MBIVC}-\sum_{U}\left(X^{T}\beta_{OLS}+e_{k}\right)$$

$$=\sum_{s}X_{s}^{T}\beta_{OLS}+\sum_{s}e_{k}+\left(\sum_{u-s}X_{k}^{T}\right){\hat{\beta }}_{MBIVC}-\sum_{U}X^{T}\beta_{OLS}-\sum_{U}e_{k}$$

$$=\sum_{s}X_{s}^{T}\beta_{OLS}+\left(\sum_{u-s}X_{k}^{T}\right){\hat{\beta }}_{MBIVC}-\sum_{U}X^{T}\beta_{OLS}-\sum_{U}e_{k}+\sum_{s}e_{k}$$

$$=-\sum_{U}X^{T}\beta_{OLS}+\sum_{s}X_{s}^{T}\beta_{OLS}+\left(\sum_{u-s}X_{k}^{T}\right){\hat{\beta }}_{MBIVC}-\sum_{U}e_{k}+\sum_{s}e_{k}$$

$$=-\sum_{U-s}X_{s}^{T}\beta_{OLS}+\left(\sum_{u-s}X_{k}^{T}\right){\hat{\beta }}_{MBIVC}-\sum_{U-s}e_{k}$$

$$=\sum_{U-s}\left(X_{s}^{T}\right)\left({\hat{\beta }}_{MBIVC}-\beta_{OLS}\right)-\sum_{U-s}e_{k}$$

Taking expected value on both sides

$$E\left({\hat{t}}_{IVMBC}-t_{y}\right)=E\left[\sum_{U-s}\left(X_{s}^{T}\right)\left({\hat{\beta }}_{MBIVC}-\beta_{OLS}\right)-\sum_{U-s}e_{k})\right]$$

$$=\sum_{U-s}\left(X_{s}^{T}\right)E\left({\hat{\beta }}_{MBIVC}-\beta_{OLS}\right)-\sum_{U-s}E\left(e_{k}\right)$$

$$=\sum_{U-s}\left(X_{s}^{T}\right)E\left({\hat{\beta }}_{MBIVC}-\beta_{OLS}\right)$$

Hence

$$Bias\left({\hat{t}}_{MBIVC}\right)=E\left({\hat{t}}_{MBIVC}-t_{y}\right)=\sum_{U-s}\left(X_{s}^{T}\right)E\left({\hat{\beta }}_{MBIVC}-\beta_{OLS}\right)$$

Proof of Theorem 4 The model-based instrumental-variable calibration estimator ${\hat{t}}_{MBIVC}$ is given by

$${\hat{t}}_{MBIVC}=\sum_{s}y_{k}+\left(\sum_{u-s}X_{k}^{T}\right){\hat{\beta }}_{MBIVC}$$

Subtracting $t_{y}$ on both sides

$${\hat{t}}_{IVC}-t_{y}=\sum_{s}y_{k}+\left(\sum_{u-s}X_{k}^{T}\right){\hat{\beta }}_{MBIVC}-t_{y}$$

$${\hat{t}}_{IVC}-t_{y}=\sum_{U-s}\left(X_{s}^{T}\right)\left({\hat{\beta }}_{MBIVC}-\beta_{OLS}\right)$$

$$=\sum_{U-s}X_{k}^{T}{\left(\sum_{s}Z_{k}^{T}X_{k}\right)}^{-1}\sum_{s}Z_{k}^{T}y_{s}-\beta_{OLS})$$

$$=\sum_{U-s}X_{k}^{T}{\left(\sum_{s}Z_{k}^{T}X_{k}\right)}^{-1}\left\{\sum_{s}(z_{k}\left(y_{k}-X_{k}\beta_{OLS}\right)\right\}$$

Taking square on both sides

$${\left({\hat{t}}_{MBIVC}-t_{y}\right)}^{2}=(\sum_{U-s}X_{k}^{T}{\left(\sum_{s}Z_{k}^{T}X_{k}\right)}^{-1}\left(\sum_{s}(z_{k}\left(y_{k}-X_{k}\beta_{OLS}\right){\left(y_{k}-X_{k}\beta_{OLS}\;\right)}^{T}z_{k}^{T}\right)$$

$${\left(\sum_{s}Z_{k}^{T}X_{k}\right)}^{-1}\left(\sum_{s}X_{k}\right)$$

Taking expected value on both sides

$$MSE\left({\hat{t}}_{MBIVC}\right)=E{\left({\hat{t}}_{MBIVC}-t_{y}\right)}^{2}=E\left[(\sum_{U-s}X_{k}^{T}{\left(\sum_{s}Z_{k}^{T}X_{k}\right)}^{-1}\left(\sum_{s}(z_{k}\left(y_{k}-X_{k}\beta_{OLS}\right){\left(y_{k}-{\;X}_{k}\beta_{OLS}\;\right)}^{T}z_{k}^{T}\right){\left(\sum_{s}Z_{k}^{T}X_{k}\right)}^{-1}\left(\sum_{s}X_{k}\right)\right]\text{.}$$
